# Vacuum-field-induced THz transport gap in a carbon nanotube quantum dot

**DOI:** 10.1038/s41467-021-25733-x

**Published:** 2021-09-16

**Authors:** F. Valmorra, K. Yoshida, L. C. Contamin, S. Messelot, S. Massabeau, M. R. Delbecq, M. C. Dartiailh, M. M. Desjardins, T. Cubaynes, Z. Leghtas, K. Hirakawa, J. Tignon, S. Dhillon, S. Balibar, J. Mangeney, A. Cottet, T. Kontos

**Affiliations:** 1 Laboratoire de Physique de l’Ecole normale supérieure, ENS, Université PSL, CNRS, Sorbonne Université, Université Paris-Diderot, Sorbonne Paris Cité, Paris, France; 2 Institute of Industrial Science and Institute for Nano Quantum Information Electronics, University of Tokyo, 4-6-1 Komaba, Meguro-ku, Tokyo, 153-8505 Japan

**Keywords:** Electronic properties and materials, Single photons and quantum effects

## Abstract

The control of light-matter interaction at the most elementary level has become an important resource for quantum technologies. Implementing such interfaces in the THz range remains an outstanding problem. Here, we couple a single electron trapped in a carbon nanotube quantum dot to a THz resonator. The resulting light-matter interaction reaches the deep strong coupling regime that induces a THz energy gap in the carbon nanotube solely by the vacuum fluctuations of the THz resonator. This is directly confirmed by transport measurements. Such a phenomenon which is the exact counterpart of inhibition of spontaneous emission in atomic physics opens the path to the readout of non-classical states of light using electrical current. This would be a particularly useful resource and perspective for THz quantum optics.

## Abstract (web summary)

Strong light-matter coupling has been realized at the level of single atoms and photons throughout most of the electromagnetic spectrum, except for the THz range. Here, the authors report a THz-scale transport gap, induced by vacuum fluctuations in carbon nanotube quantum dot through the deep strong coupling of a single electron to a THz resonator.

## Introduction

There are now many examples of light-matter interfaces coupling coherently single atoms (real or artificial) and single photons throughout most of the electromagnetic spectrum from the microwave to optical regions. A notable exception is the terahertz range for which only collective modes of matter have very recently been coupled ultra-strongly to light^1,2^. Coupling strongly an individual charge dipole to light requires the use of the electrical component of light^3^. Despite the fact that the electrical coupling is very often described by a dipole-electric field coupling, it is important to stress that the microscopic origin of this phenomenon is a coupling of the electron density to the electric field of the form^4^:

1 $${H}_{dot-cavity}=e\int {d}^{3}r\hat{\rho }(r)v(r){V}_{rms}(a+{a}^{{{{\dagger}}} })$$

where $\hat{\rho }(r)$ is the electronic density, *v*(*r*) the distribution function of the electric field and *V*_*rms*_(*a* + *a*^†^) is the quantized electric field corresponding to a cavity mode of frequency *f*_*cav*_, with *V*_*rms*_ the amplitude of the vacuum fluctuations. The above expression bridges circuit QED and cavity QED setups. Since it is derived from the minimal gauge invariant form of the light-matter hamiltonian, it contains all possible electrical coupling schemes. The density operator can be written using the general expression $\hat{\rho }(r)={{{\Psi }}}^{{{{\dagger}}} }(r){{\Psi }}(r)$, where Ψ(*r*) is the field operator of the particles (electrons or holes) interacting with the electromagnetic mode.

The special case of one electron shared between two electronic orbitals allows one to recast the two important forms, only apparently different, of electric coupling schemes. Defining two orbital wave functions *ϕ*_1(2)_(*r*) and annihilation operators *c*_1(2)_, we can write Ψ(*r*) = *ϕ*_1_(*r*)*c*_1_ + *ϕ*_2_(*r*)*c*_2_. The coupling hamiltonian *H*_*dot*−*cavity*_ takes the simple form:

2 $${H}_{dot-cavity}=(2\pi {g}_{l}\hat{n}+2\pi {g}_{t}{\hat{\sigma }}_{x})(a+{a}^{{{{\dagger}}} })$$

where $\hat{n}={c}_{1}^{{{{\dagger}}} }{c}_{1}+{c}_{2}^{{{{\dagger}}} }{c}_{2}$ and ${\hat{\sigma }}_{x}={c}_{1}^{{{{\dagger}}} }{c}_{2}+{c}_{2}^{{{{\dagger}}} }{c}_{1}$ (we have assumed, for simplicity, that the two orbitals couple to the field with the same strength). The first term is the longitudinal coupling of the Franck-Condon^5–7^ problem familiar to the field of quantum dots and molecules coupled to quantized vibrations or, more generally, optomechanics^8,9^. The second term is the transverse coupling, as presented in the quantum optics field and in particular to those studies exploring the ultra-strong coupling between light and matter. However, both terms coexist in general and are expected to lead to strong modifications of both light and matter when the g factors are comparable to the cavity frequency. One can characterize the strength of the coupling by dividing *g*_*l*/*t*_ by the bare frequency of the electromagnetic mode *f*_*cav*_. We define the dimensionless parameters $\tilde{g}={g}_{l}/{f}_{cav}$ and $\bar{g}={g}_{t}/{f}_{cav}$.

**Fig. 1:**
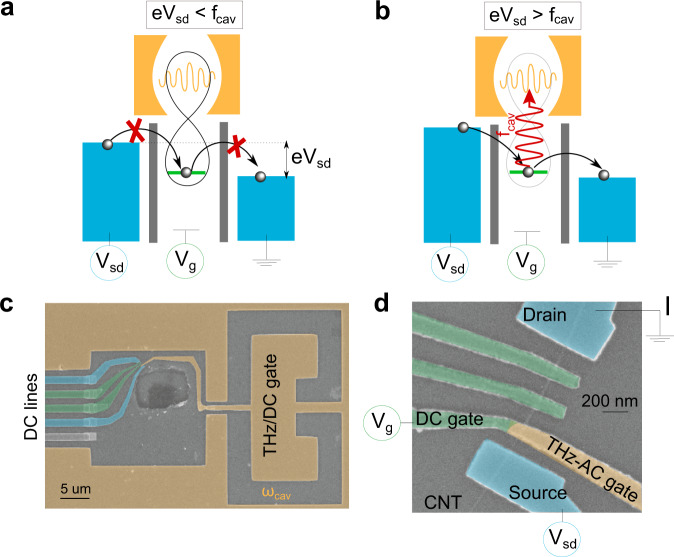
Device geometry and working principle. **a** Cartoon picture of the process leading to the gap. At low bias with respect to the cavity frequency *f*_*cav*_, the light and matter hybridize ultra-strongly, leading to a blockade of the current even though the cavity is in its vacuum state. **b** Cartoon picture of the process leading to the gap. At high bias with respect to the cavity frequency *f*_*cav*_, the light and matter are not hybridized anymore and the current is de-blocked via photon emission processes (*n* = 1 process sketched here). The process a. illustrates the appearance of a band gap induced by the vacuum fluctuation of the cavity. **c** SEM picture of the devices: the THz cavity is capacitively coupled to the QD in the CNT. **d** Close-up of a similar device showing contacts and top gates on the CNT. The gate layout is slightly different than for panel **c**. I is the current flowing through the device.

In the case of strong hybridization between light and matter, one expects changes in the electronic transport properties. This has recently attracted considerable interest in the limit of many particles or collective modes^10–15^. In these works, the large particle density helps to reach strong light-matter hybridization. This contrasts with a single electron occupying a single orbital that we study here. It can be recast with the above paradigm. The orbital of the dot plays the role of orbital 1 and the orbitals of the reservoir electrons play the role of orbital 2 (for simplicity, the $\bar{g}$ term can be disregarded due to the weak overlap between the dot orbital and the reservoir orbitals). The dipoles sketched in Fig. 1a, b are thus tunneling dipoles. In such a setup, it has been predicted that a band gap equal to the energy of the mode can emerge^6^ when $\tilde{g}\approx 1$, a regime called deep-strong coupling regime. This situation can be understood with a simple tunneling argument. As sketched in Fig. 1a, for strong hybridization of light and matter, the matter part of the wave function hybridizes with that of free electrons as long as the energy of the electrons is smaller than the energy of the mode^5–7^. For $\tilde{g}\approx 1$, a strong suppression of the conductance below the photon energy is predicted. Interestingly, this phenomenon should occur even when the cavity is completely in the vacuum state of the electromagnetic field. This is a result of quantum fluctuations of the cavity field which continuously “push” the electronic wave function away from the resonant conditions that permit electronic transport. Such a phenomenon is optimum until tunneling events start to emit 1, 2,... n photons into the cavity. Each multi-photon process contributes to ${\tilde{g}}^{n}$ to the current and peaks at *eV*_*sd*_ = *nhf*_*cav*_ (see Methods). The *n* = 1 process is sketched in Fig. 1b. Below this threshold, the conductance is predicted to be strongly suppressed^6,7^. This explains qualitatively the prediction of a gap in the matter spectrum and can be seen as the counterpart of inhibition of spontaneous emission in atomic physics. Note, however, that in a longitudinal coupling scheme described above, we do not expect a normal mode splitting like in the conventional case as the corresponding coupling term (with prefactor *g*_*l*_) commutes with the unperturbed quantum dot hamiltonian. Finally, it is worth mentioning that the internal dot-dot dipoles corresponding to the transverse coupling *g*_*t*_ correspond a priori to higher energies than those of the resonator (in the several THz ranges). Therefore, we do not expect to be sensitive to the transverse coupling in our experiment, as specified above, although the two coupling schemes should be of the same order of magnitude.

Here, we directly observe such a phenomenon by embedding a quantum dot in a THz resonator. By engineering a deep-strong coupling between a single electron trapped in the carbon nanotube quantum dot and the THz cavity, we induce an energy gap of 2.6 meV ≈ 0.6 THz that is directly visible in the conductance of the electronic system. Such a gap is observed in 3 different devices all realised with a similar nanolithography layout. The suppression of the conductance within ±2.6 mV allows us to extract $\tilde{g}\;\gtrsim\; 1$, placing our single electron system in the deep-strong coupling regime.

Opening a vacuum field-induced THz gap requires building an experimental system with stringent requirements. First, one has to isolate a single electron with confinement energy in the THz range. Second, one has to engineer an optical mode highly focused on the electronic wave function. Carbon nanotubes are a particularly well suited material as they can form clean quantum dots with energy level spacing Δ*E* naturally lying in the THz range for system dimensions of a few hundreds of nanometres^16^. They are also particularly well suited to be coupled to the electromagnetic field as recently demonstrated in microwave measurements^17,18^.

## Results

**Fig. 2:**
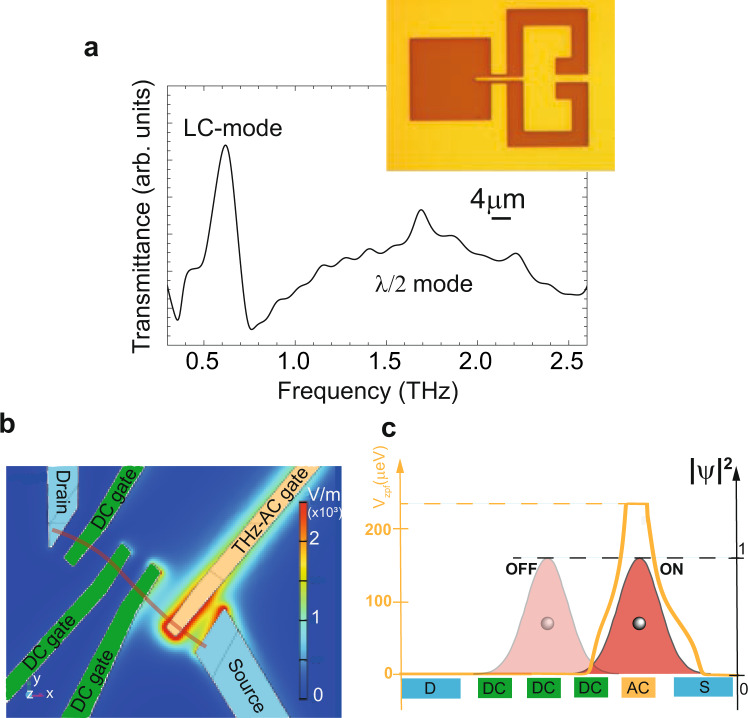
Cavity characterisation, device geometry and working principle. **a** Transmittance spectrum of the cavity measured on a reference ensemble, revealing the LC-mode of the cavity at 0.62 THz and a much broader dipolar mode at ca 1.75 THz. Inset: Cavity geometry **b** Simulation of the electric field distribution at resonance, showing how the LC-mode of the cavity couples to the QD and the strong focusing of the cavity mode on the QD. The LC-mode is found to be at 0.54 GHz, close to the experimental measurement of panel **a**. **c** Microwave simulations enriched by a sketch of the electronic wave function in the “ON” and “OFF” regime explaining how the coupling strength depends on the gate voltage setting of the devices.

Our experimental setup is depicted in Fig. 2. The QDs are realised by transferring the CNTs into the lateral square of our cavity and realising the source/drain contacts and the top gates. One of the latter is connected to the “finger” coming from a Split Ring Resonator(SRR) (see Fig. 1c, d for an example of such device, more details in the methods) and can act both as DC- and AC-gate (see Fig. 1d), since the THz does not propagate through the finger. The THz cavity, shown in the inset of Fig. 2a), is an optimised version of a well-established THz SRR, widely investigated in the metamaterial community^19–23^ and already used for various experiments^24–26^. This LC-mode was characterised via transmission measurements with terahertz time-domain spectroscopy (TDS) at low-temperatures ( ≈10*K*) on a separate sample containing an array of the designed cavities: the LC-mode has a frequency of 0.62 THz and a quality factor of about 5, as shown in Fig. 2a. This is consistent with finite-elements simulations (COMSOL Multiphysics) which allow us to plot the mode profile (see Fig. 2b).

**Fig. 3:**
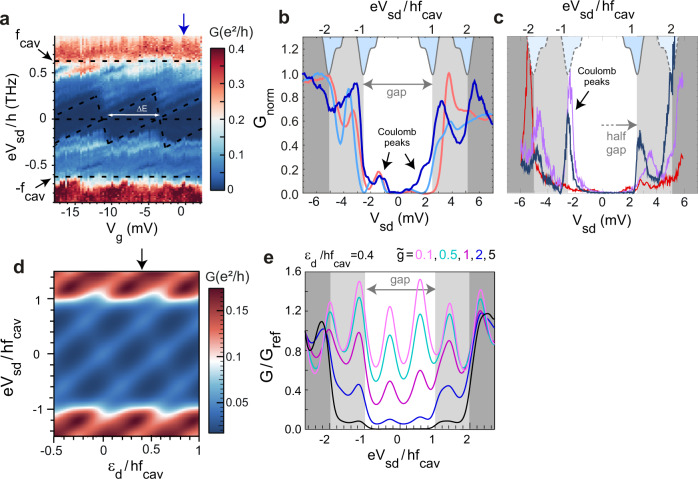
Terahertz gap in the dc-transport of the devices. **a** Conductance *G* map of device A as a function of *V*_*g*_ and *V*_*sd*_. Coulomb diamonds are visible in the map. They appear inside the region highlighted by black dashed lines at the cavity frequency. The blue vertical arrow indicates the cut represented in panel (**b**). **b** Normalised conductance *G* as a function of bias *V*_*sd*_ for several gate voltages for samples A and B. The dark blue line is for sample A, *Vg* = −0.25 mV, the light blue line is for sample B, *Vg* = −8.90 mV, and the orange line is for sample B, *Vg* = −11.3 mV. The topmost (inverted) gray curves are the LC-mode resonance centered at ± *f*_*cav*_ and ± 2*f*_*cav*_. **c** Normalised conductance *G* as a function of bias *V*_*sd*_ for several gate voltages for sample C. The dark blue line is for *Vg* = 42 mV, the pink line is for *Vg* = 47.5 mV and the red line is for *Vg* = 69.5 mV. **d** Colorscale plot of the theoretical conductance map reproducing qualitatively that of panel (**a**). **e** Linear cut of the conductance as predicted from theory and showing the gap edge at ± *f*_*cav*_ and the two-photon conductance step at ± 2*f*_*cav*_ observed for sample A (see parameters in the methods), for increasing dimensionless coupling strength $\tilde{g}$.

Our main findings are presented in Fig. 3 and correspond to three different devices, each coupling a THz cavity to CNT-QDs, all essentially identical. The clearest conductance map is presented in Fig. 3a. The conductance at large bias is large for a quantum dot, of the order of 0.4 × *e*^2^/*h*, whereas there is a stripe of strong suppression at low bias where the conductance stays roughly below 0.05 × *e*^2^/*h*. This signals a clear gap in the spectrum of the quantum dot from about −2.6 mV (0.63 THz) to +2.6 mV throughout the plotted region. Faintly visible in light blue (below 0.05 × *e*^2^/*h*) is the remainder of the conductance of the uncoupled electronic states which would constitute the unperturbed coulomb diamonds (highlighted by black dashed lines). The normalised conductance *G*_*norm*_ vs source-drain bias at fixed gate voltage is shown in Fig. 3b, c (vertical cut of the map in Fig. 3a), along with representative traces of the gaps in the other samples. It is striking that all gap edges approximately collapse onto each other and correspond to the energy of the cavity mode, as calibrated from the direct THz transmission measurements. For samples A and B, the gap is symmetric and both positive and negative gap edges are visible as shown in figure Fig. 3b. Sample B can also display negative differential resistance after the gap edge (see Supplementary Note 1) which we attribute to interaction effects and multiple levels participating to transport at large bias. However, the suppressions of conductance for Sample B and Sample A below ±2.6 mV are qualitatively the same as one can see from Fig. 3b comparing the dark blue and orange curves (samples A and B respectively). For sample C, only the positive gap edge is visible as shown in figure Fig. 3c (see Supplementary Note 1 for the full conductance maps of samples A, B, and C). We attribute this effect to the asymmetry of tunneling between the left and the right contact, which can be quantified by Γ_*L*_ and Γ_*R*_ in the tunneling model shown in the methods. Importantly, devices B and C have also large maximum conductances (respectively 0.3 × *e*^2^/*h* and 1.7 × *e*^2^/*h*). This means that below the energy corresponding to the cavity frequency, the quantum dots, initially well coupled to the leads, become essentially decoupled from the leads on a wide gate voltage range. In order to get further insights on our findings, we use a theory based on non equilibrium Green’s functions^7^. We use that theoretical approach rather than a master equation^6^ since our experiments are carried out in the regime Γ_*L*,*R*_ ≫ *k*_*B*_*T*, where *T* is the temperature. It is interesting to note that the theory, presented in Fig. 3e is able to reproduce qualitatively the observed gap as well as the two-photon peak present for sample A at twice the cavity frequency. As shown in Fig. 3e, the theory also predicts asymmetries in the gap edges but they are not as strong as in the data of sample C, which we attribute to our mean-field treatment for the electron-electron interactions. Nevertheless, the theory is also able to reproduce qualitatively the 2D color scale plot as illustrated for sample A. In particular, the gap edge is highlighted by the transition from blue to red color of the conductance map, as shown in Fig. 3d. It seems that the overall curve better matches for $\tilde{g}\approx 2$ than for higher $\tilde{g}$ (we show that $\tilde{g}=5$ is markedly different from our findings for the overall shape, although the gap is more pronounced than for lower coupling values). This confirms our estimate of deep strong coupling with $\tilde{g}\;\gtrsim\; 1$.

The presence of such a gap in the transport characteristics of the dot is a qualitative signature that the normalised coupling strength between the electronic level and the THz mode $\tilde{g}$ is greater or equal to unity. It was first predicted by Koch, Von Oppen, and Andreev^6^ in the context of the Franck-Condon blockade where a single quantum dot is deep-strongly coupled to a vibrational mode. The form of the electron-boson coupling used in this reference is in fact very general and has been the subject of many theoretical works. This configuration directly maps to our problem by simply replacing the vibrational mode by an electromagnetic mode. The theory predicts that the conductance should be exponentially suppressed as ${e}^{-{\tilde{g}}^{2}}$ with respect to the high bias conductance (see equation (27) in Methods). Interestingly, the theory presented in the methods section also predicts the step height for the first step as a function of $\tilde{g}$, *f*_*cav*_ and Γ_*L*/*R*_. From the traces in Fig. 3b, the ratio between the gap edge height and that of the subgap conductance peaks can be estimated conservatively to be about 5 for samples A and B. From the theory, this gives $\tilde{g}\;\gtrsim\; 1$ which is close to the value stated above. The investigated systems are therefore in the deep strong coupling regime with an estimated coupling strength comparable to the best ones reported up to date in the many-particle regime^25^. In addition, our system involves a single electronic transition and not an ensemble as presented until now in the literature. Using Γ_*L*/*R*_ ≈ 1.2 meV and 0.9 meV extracted from the full width at half maximum of the Coulomb peaks for sample A and B, we can then derive cooperativity of $C\approx 2{\tilde{g}}^{2}\times {f}_{cav}^{2}/\kappa {{\Gamma }}\;\gtrsim\; 50$ for the single electron, where *κ* is the half-width at half maximum of the cavity measured by TDS to be 2*π* × 0.06 THz and Γ = Γ_*L*_ = Γ_*R*_ is the width of the conductance peaks in the symmetric case. In this respect, one should note that the optical mode energy of 2.6 meV corresponds to a mode temperature of 30 K. The measurements were performed at temperatures between 50 and 300 mK, in the dark. The optical mode is therefore in its ground state (thermal occupation *n*_*therm*_ < <1) and only photons available are provided by the vacuum fluctuations.

## Discussion

Why can our system be placed in the deep-strong coupling regime? As shown in equation (1), the main features which set the magnitude of the coupling strength are the zero-point fluctuation voltage of the field *V*_*rms*_ and the overlap between the wave function of the electron and the photonic pseudo-potential distribution *v*(*r*) characterized by the integral $\tilde{v}=\int {d}^{3}r\rho (r)v(r)$ (see equation (1)). In our case, the AC-electric potential is strongly focused onto the dot owing to the AC top gate as shown in the electromagnetic simulations of Fig. 2b, c, similar to GHz experiments^17,18^. This leads to $\tilde{v}\approx 1$, in contrast to the other THz investigations where $\tilde{v}\ll 1$, as the full vacuum fluctuation voltage of the cavity field can be coupled to the electronic orbitals of the dot. Interestingly, the electromagnetic simulation shows that without the nanotube, the zero-point fluctuations of the cavity electric potential can be very large, of about 200−400 μeV. In such conditions, the nanotube and the cavity cannot be considered separately and the matter system is expected to strongly modify the geometry of the mode. Electron-electron correlations play a crucial role in screening processes and our findings suggest that they could boost $\tilde{g}$ further into the deep strong coupling regime. Finally, it is interesting to note that $\tilde{v}$ is a priori gate voltage-dependent which can tune the wave function position as sketched in Fig. 2c. In the “ON” state, we have $\tilde{v}\approx 1$ whereas in the “OFF” state, we have $\tilde{v}\ll 1$.

Our system is a promising platform to implement quantum optical protocols and experiments in the THz range using solid-state devices. First, it could generally enable the direct investigation of the light-matter interaction in the non-perturbative limit^27,28^ and of recently predicted effects such as ground state luminescence^29^. It could also be used to study the interplay between Coulomb blockade and quantum properties of the light in the cavity, e.g. its squeezing^30,31^. Additionally, it can be interesting to use the longitudinal coupling for charge read-out in the THz range by mixing the cavity field with free-space coherent THz light as recently proposed in the GHz range for qubit readout. Finally, it is interesting to note that the theory, for example in formula (22), predicts that each Fock state present in the vacuum field should have a specific contribution to the current. This suggests that our setup can be used as a photon number resolved detector (The value of the Γ’s would need to be reduced in order to better resolve each Fock state peak). One can also anticipate from this analogy that the full Wigner function of the field could be reflected in higher-order moments of the current. In this case, this approach would be useful for detecting any non-classical states of THz light.

## Methods

### Experimental

The THz-SRRs were realised via electron-beam lithography (EBL) and reactive-ion etching onto a 100–150 nm layer of Nb evaporated onto 500 *nmSiOx*/500 *μm* high-resistivity Si substrate. The dimensions of the ground plane opening for the SRR are 20 μm × 34 μm. The size of the gap is 4 μm (see Fig. 1a). Our CNTs are grown by chemical vapor deposition from *H*_2_ and *CH*_4_ onto a separate quartz chip and then stamped to the sample area mentioned in the main text. They are localized with respect to alignment markers with a scanning electron microscope. The nanotubes used in this study where metallic or small bandgap semiconducting single-wall carbon nanotubes as characterized from transport measurements. Our growth recipe predominantly produces single-wall carbon nanotubes with a low density of bundles. The contacts and gates are designed by EBL followed by e-beam evaporation and lift-off of a 70 nm-thick Pd layer and of a 5 nm oxidised Al layer capped by a 40/20 nm thick Al/Pd layers, respectively. Current and conductance are measured simultaneously in the samples with a trans-impedance amplifier, with gain 10^7^ V/A, a DC-source, and a lock-in amplifier. Samples B and C were measured in a ^3^*He* cryostat (base temperature 230 mK), sample A in a dilution refrigerator (at 50 mK).

We modify the layout of the SRRs by opening a square on the side (size 20 μm × 20 μm), where we transfer the CNT to realise the QDs. From the middle of the capacitor of the SRR, where the electric field of the main (LC) resonance is concentrated, a finger protrudes to the QD area. Such modifications keep the general properties of the SRR intact, in the same way as done for other SRR-based optoelectronic devices. Electrically, the conductive plane in which the SRR is etched acts as a ground plane and keeps the QD protected from stray currents and fields. As discussed in the literature^20,21^, such cavity is linearly polarised and the LC-mode is the lowest in energy and the one with the best quality factor (hence we disregard the higher-order more delocalised modes in our analysis).

### Non equilibrium Green’s function theory for the vacuum field gap

In this section, we present the theoretical description of photon-assisted transport through a multi-orbital quantum dot, which we have used to interpret our data. Many references have used a sequential tunneling description of electronic transport, which is valid for a temperature much larger than the tunneling rates between the dot and its normal metal reservoirs. However, our experiment seems to be in the opposite regime since the width of the conductance peaks in our data is much larger than the temperature. This is why we have chosen to use a description of electronic transport that uses the non equilibrium Keldysh Green’s functions of a multi-orbital quantum dot. Related approaches for the single orbital case were presented in refs. ^32,33^.

We present here a first approach intended to capture the essence of our observations. For simplicity, we disregard the effect of Coulomb repulsion in the dot. In the deep Coulomb blockade regime which is observed in our samples, this should essentially lead to qualitative differences in the *I* − *V* curves, like for instance a shift in the position of the resonant lines in the conductance maps versus bias and gate voltages. A division by 2 of the amplitude of the conductance peaks also occurs due to Coulomb blockade, but this can be mimicked in the non-interacting case by using a spinless model. Hence, we have chosen to use the spinless Hamiltonian of a quantum dot connected to two spinless fermionic reservoirs *L* and *R* and coupled to a microwave cavity with frequency *f*_*cav*_, which we write:

3 $$\hat{H}={\hat{H}}_{el}+{\hat{H}}_{cav}+\mathop{\sum}\limits_{d}2\pi {g}_{l}({\hat{a}}^{{{{\dagger}}} }+\hat{a}){\widehat{c}}_{d}^{{{{\dagger}}} }{\widehat{c}}_{d}$$

with

$${\hat{H}}_{el}=\mathop{\sum}\limits_{d}{\varepsilon }_{d}{\widehat{c}}_{d}^{{{{\dagger}}} }{\widehat{c}}_{d}+\mathop{\sum}\limits_{k}{\varepsilon }_{k}{\hat{c}}_{k}^{{{{\dagger}}} }{\hat{c}}_{k}+\mathop{\sum}\limits_{k,d}({V}_{kd}^{* }{\widehat{c}}_{d}^{{{{\dagger}}} }{\hat{c}}_{k}+{V}_{kd}{\hat{c}}_{k}^{{{{\dagger}}} }{\widehat{c}}_{d}^{{{{\dagger}}} })$$

and

4 $${\hat{H}}_{cav}=2\pi {f}_{cav}{\hat{a}}^{{{{\dagger}}} }\hat{a}+\mathop{\sum}\limits_{\beta }{\omega }_{\beta }{b}_{\beta }^{{{{\dagger}}} }{b}_{\beta }+{U}_{\beta }({\hat{a}}^{{{{\dagger}}} }+\hat{a})({b}_{\beta }^{{{{\dagger}}} }+{b}_{\beta })$$

Above, *ε*_*d*_ is the orbital energy of the dot level *d* with creation operator ${\widehat{c}}_{d}^{{{{\dagger}}} }$, and *ε*_*k*_ is the orbital energy of the level *k* with creation operator ${\widehat{c}}_{k}^{{{{\dagger}}} }$ in the fermionic reservoir *L* or *R*. We denote by *V*_*kd*_ the tunnel hoping constant between orbitals *d* and *k*, ${\hat{a}}^{{{{\dagger}}} }$ the creation operator for a cavity photon. *U*_*β*_ is the coupling constant between the cavity and the modes *β* of the cavity damping bath with creation operators ${b}_{\beta }^{{{{\dagger}}} }$. Finally, *g*_*l*_ is the longitudinal coupling between the dot and the cavity electric field. We disregard the other types of light-matter couplings which we expect to be much weaker. To understand the effect of the light-matter coupling *g*_*l*_, it is convenient to perform a polaron transformation^6^, such that for any operator $\hat{A}$, one has:

5 $$\hat{{{{{{{{\bf{A}}}}}}}}}={e}^{S}\hat{A}{e}^{-S}$$

with

6 $$S=\mathop{\sum}\limits_{d}\tilde{g}({a}^{{{{\dagger}}} }-a){\widehat{c}}_{d}^{{{{\dagger}}} }{\widehat{c}}_{d}$$

and

7 $$\tilde{g}=\frac{{g}_{l}}{{f}_{cav}}$$

In this framework, the operators ${\widehat{c}}_{d}$ and $\hat{a}$ are transformed into

8 $${\widehat{{{{{{{{\bf{c}}}}}}}}}}_{{{{{{{{\bf{d}}}}}}}}}={\widehat{c}}_{d}\hat{X}$$

and

9 $$\hat{{{{{{{{\bf{a}}}}}}}}}=\hat{a}-\frac{{g}_{l}}{{f}_{cav}}\mathop{\sum}\limits_{d}{\widehat{c}}_{d}^{{{{\dagger}}} }{\widehat{c}}_{d}$$

with $\hat{X}=\exp (i\tilde{g}\hat{P})$ and $\hat{P}=-i(\hat{a}-{\hat{a}}^{{{{\dagger}}} })$. The system Hamiltonian $\hat{H}$ is transformed into:

10 $$\hat{{{{{{{{\bf{H}}}}}}}}}=\mathop{\sum}\limits_{d}{\bar{\varepsilon }}_{d}^{{{{\dagger}}} }{{\widehat{{{{{{{{\bf{c}}}}}}}}}}_{d}^{{{{\dagger}}} }\widehat{c}}_{{{{{{{{\bf{d}}}}}}}}}+\mathop{\sum}\limits_{k}{\varepsilon }_{k}{\hat{c}}_{k}^{{{{\dagger}}} }{\hat{c}}_{k}+\mathop{\sum}\limits_{k,d}({V}_{kd}^{* }{\widehat{{{{{{{{\bf{c}}}}}}}}}}_{{{{{{{{\bf{d}}}}}}}}}^{{{{\dagger}}} }{\hat{c}}_{k}+{V}_{kd}{\hat{c}}_{k}^{{{{\dagger}}} }{\widehat{{{{{{{{\bf{c}}}}}}}}}}_{{{{{{{{\bf{d}}}}}}}}})+{\hat{H}}_{cav}$$

with ${\bar{\varepsilon }}_{d}={\varepsilon }_{d}-\frac{2\pi {g}_{l}^{2}}{{f}_{cav}}$ and ${\hat{H}}_{cav}$ defined by Eq. (4). Above, the effect of *S* on the terms in *U*_*β*_ is disregarded, which is justified for small enough values of *U*_*β*_ (see ref. ^33^). We also disregard interaction terms in $-\frac{2\pi {g}_{l}^{2}}{{f}_{cav}}{\widehat{c}}_{d}^{{{{\dagger}}} }{\widehat{c}}_{d}{\widehat{c}}_{{d}^{\prime}}^{{{{\dagger}}} }{\widehat{c}}_{{d}^{\prime}}$ for the same reason as we disregard Coulomb interactions: these terms will essentially shift the position of the resonant conductance lines in the gate-voltage/bias-voltage two-dimensional maps. Since the operators ${\hat{c}}_{k}$ related to the states in the electronic reservoirs are unchanged when the transformation *S* is applied (${\hat{{{{{{{{\bf{c}}}}}}}}}}_{k}={\hat{c}}_{k}$), one can calculate the current through the dot as ${I}_{C}=-e\frac{d}{dt}\mathop{\sum}\limits_{k}{\left\langle {\hat{c}}_{k}^{{{{\dagger}}} }{\hat{c}}_{k}\right\rangle }_{\hat{{{{{{{{\bf{H}}}}}}}}}}$ where ${\left\langle \right\rangle }_{\hat{{{{{{{{\bf{H}}}}}}}}}}$ denotes the statistical average for a system whose dynamics is described by $\hat{{{{{{{{\bf{H}}}}}}}}}$. For simplicity, we assume that the hoping constant *V*_*kd*_ is independent from *d* and just depends on whether the level *k* belongs to the left or right reservoir (*V*_*kd*_ = *V*_*L*(*R*)_ for *k* ∈ *L*(*R*)). In this case, one can define tunnel rates ${{{\Gamma }}}_{L(R)}=2\pi {|{V}_{L(R)}|}^{2}{\rho }_{L(R)}$, with *ρ*_*L*(*R*)_ the densities of states in the L(R) reservoirs. Since $\{{\widehat{{{{{{{{\bf{c}}}}}}}}}}_{{{{{{{{\bf{d}}}}}}}}}^{{{{\dagger}}} },{\widehat{{{{{{{{\bf{c}}}}}}}}}}_{{{{{{{{\bf{d}}}}}}}}}\}=1$, we will use the general expression^32,33^

11 $$I=\frac{e}{\hslash }i\int \frac{d\omega }{2\pi }\frac{{{{\Gamma }}}_{L}{{{\Gamma }}}_{R}}{{{{\Gamma }}}_{L}+{{{\Gamma }}}_{R}}({f}_{L}(\omega )-{f}_{R}(\omega ))\mathop{\sum}\limits_{d}\left({{{{{{{{\mathcal{G}}}}}}}}}_{d}^{ \,{ > }\,}(\omega )-{{{{{{{{\mathcal{G}}}}}}}}}_{d}^{\,{ < }\,}(\omega )\right)$$

to express the current *I* through the dot, with

12 $${{{{{{{{\mathcal{G}}}}}}}}}_{d}^{\,{ < }\,}(t)=i{\left\langle {\widehat{{{{{{{{\bf{c}}}}}}}}}}_{{{{{{{{\bf{d}}}}}}}}}^{{{{\dagger}}} }(0){\widehat{{{{{{{{\bf{c}}}}}}}}}}_{{{{{{{{\bf{d}}}}}}}}}(t)\right\rangle }_{\hat{{{{{{{{\bf{H}}}}}}}}}}$$

and

13 $${{{{{{{{\mathcal{G}}}}}}}}}_{d}^{ \,{ > }\,}(t)=-i{\left\langle {\widehat{{{{{{{{\bf{c}}}}}}}}}}_{{{{{{{{\bf{d}}}}}}}}}(t){\widehat{{{{{{{{\bf{c}}}}}}}}}}_{{{{{{{{\bf{d}}}}}}}}}^{{{{\dagger}}} }(0)\right\rangle }_{\hat{{{{{{{{\bf{H}}}}}}}}}}$$

Note that, strictly speaking, Eq. (11) is exact only in the absence of coupling to the cavity^34,35^ ($\tilde{g}=0$). In principle, due to the dressing of the ${\widehat{{{{{{{{\bf{c}}}}}}}}}}_{{{{{{{{\bf{d}}}}}}}}}$ term by $\hat{X}$ and due to the term ${\hat{H}}_{cav}$ in (10), some extra contributions should be added to Eq. (11). However, the cavity has a dissipation rate that is much smaller than the one of the electronic system, and thus a much slower evolution. In this limit, one can perform a Born-Oppenheimer approximation which consists in calculating the electronic circuit dynamics independently from the one of the cavity, with $\hat{X}$ treated as a “parameter”. This approximation, which is equivalent to disregarding the component ${\hat{H}}_{cav}$ in Eq. (10) to calculate *I*, leads to Eq. (11) for a finite electron/photon coupling ($\tilde{g}\;\ne\; 0$).

Using the definition (8), equations (12) and (13) give

14 $${{{{{{{{\mathcal{G}}}}}}}}}_{d}^{\,{ < }\,}(t)=i{\left\langle {\widehat{c}}_{d}^{{{{\dagger}}} }(0){\widehat{c}}_{d}(t){X}^{{{{\dagger}}} }(0)X(t)\right\rangle }_{\hat{{{{{{{{\bf{H}}}}}}}}}}$$

and

15 $${{{{{{{{\mathcal{G}}}}}}}}}_{d}^{ \,{ > }\,}(t)=-i{\left\langle {\widehat{c}}_{d}(t){\widehat{c}}_{d}^{{{{\dagger}}} }(0)X(t){X}^{{{{\dagger}}} }(0)\right\rangle }_{\hat{{{{{{{{\bf{H}}}}}}}}}}$$

In equation (11), the Fermi occupation functions of the states in the reservoirs *L* and *R* can be expressed as

$${f}_{L}(\omega )=\frac{1}{1+\exp [(\hslash \omega -eVx)/{k}_{B}T]}$$

and

$${f}_{R}(\omega )=\frac{1}{1+\exp [(\hslash \omega +eV(1-x))/{k}_{B}T]}$$

where *x* ∈ [0, 1] is a number that takes into account how the voltage drop *V* is distributed along the dot circuit, depending on the capacitances of the tunnel junctions between the dot and the reservoirs. We now use an approximation of ${{{{{{{{\mathcal{G}}}}}}}}}_{d}^{\lessgtr }(t)$ in the Born-Oppenheimer spirit, i.e.

16 $${{{{{{{{\mathcal{G}}}}}}}}}_{d}^{\,{ < }\,}(t)=i{\left\langle {\widehat{c}}_{d}^{{{{\dagger}}} }(0){\widehat{c}}_{d}(t)\right\rangle }_{\hat{{{{{{{{\bf{H}}}}}}}}}}{\left\langle {X}^{{{{\dagger}}} }(0)X(t)\right\rangle }_{\hat{{{{{{{{\bf{H}}}}}}}}}}$$

and

17 $${{{{{{{{\mathcal{G}}}}}}}}}_{d}^{ \,{ > }\,}(t)=-i{\left\langle {\widehat{c}}_{d}(t){\widehat{c}}_{d}^{{{{\dagger}}} }(0)\right\rangle }_{\hat{{{{{{{{\bf{H}}}}}}}}}}{\left\langle X(t){X}^{{{{\dagger}}} }(0)\right\rangle }_{\hat{{{{{{{{\bf{H}}}}}}}}}}$$

We furthermore treat the averages ${\left\langle \right\rangle }_{\hat{{{{{{{{\bf{H}}}}}}}}}}$ in the above expressions “at lowest order in $\tilde{g}$” i.e. by treating the electronic and photonic dynamics independently due to the limited value of $\tilde{g}$. In this framework, $\tilde{g}$ is involved only in the definition of the *X* and *X*^†^ operators. We thus obtain

18 $${{{{{{{{\mathcal{G}}}}}}}}}_{d}^{\,{ < }\,}(t)\simeq {G}^{\,{ < }\,}(t){\left\langle {X}^{{{{\dagger}}} }(0)X(t)\right\rangle }_{{\hat{H}}_{cav}}$$

and

19 $${{{{{{{{\mathcal{G}}}}}}}}}_{d}^{ \,{ > }\,}(t)\simeq {G}^{ \,{ > }\,}(t){\left\langle X(t){X}^{{{{\dagger}}} }(0)\right\rangle }_{{\hat{H}}_{cav}}$$

with $${G}^{\,{ < }\,}(t)=i{\left\langle {\widehat{c}}_{d}^{{{{\dagger}}} }(0){\widehat{c}}_{d}(t)\right\rangle }_{{\hat{H}}_{el}}$$ and $${G}^{ \,{ > }\,}(t)=-i{\left\langle {\widehat{c}}_{d}(t){\widehat{c}}_{d}^{{{{\dagger}}} }(0)\right\rangle }_{{\hat{H}}_{el}}$$.

It is important at this point to comment on the approximations made so far. Up to equation (17) we use the polaronic transformation which is exact and therefore is still valid in the deep strong coupling regime. The polaron transformation modifies the tunnel terms in hamiltonian (10) by dressing the coupling term with the displacement operator ${e}^{\tilde{g}(a-{a}^{{{{\dagger}}} })}$, from which stems the exponential quenching of the Γ’s and therefore the conductance in the vacuum state of the electromagnetic mode as we describe below. In order to calculate the full conductance map, one needs to treat the multiphoton processes present in the expansion of the displacement operator. This can be done essentially in a non-perturbative manner using a master equation formalism like in ref. ^6^. This limit is restricted to Γ ≪ *k*_*B*_*T* and therefore does not apply to the regime of our experiment, unfortunately. Furthermore, we would like to emphasize that we do not neglect interactions between electrons but rather treat them in a mean-field theory. As shown by Meir and Wingreen in ref. ^34^ and^35^, the equation of motion technique for non equilibrium Green’s functions which we use here is able to describe the Coulomb blockade and the renormalization of the energy levels by interactions. It can be even modified to treat strong correlation and Kondo physics. The equation of motion produces a hierarchy of correlators which must be truncated in order to get a closed set of equations. Coupling to bosons induces further complexity but ref. ^7^ and^33^ show how to do this. This implies further truncation between the fermion and boson correlators. The validity of the approximations has been studied in ref. ^7^ and^33^ to some extent and shows that the EOM can work for large Gamma, U, and g. Although our theory could include further correlation effects such as energy renormalization and Kondo physics, it is a good starting point for the analysis. We are therefore confident that the theory used in our work allows us to reliably estimate $\tilde{g}$ even in the non-perturbative regime.

For temperatures *T* much smaller than the cavity mode frequency *f*_*cav*_, the cavity correlators ${\left\langle {X}^{{{{\dagger}}} }(0)X(t)\right\rangle }_{\tilde{g} = 0}$ can be calculated for a cavity in its fundamental state as

20 $${\left\langle X(t){X}^{{{{\dagger}}} }(0)\right\rangle }_{{\hat{H}}_{cav}}={e}^{-{\tilde{g}}^{2}}{e}^{{\tilde{g}}^{2}{e}^{-i2\pi {f}_{cav}t}}={e}^{-{\tilde{g}}^{2}}\mathop{\sum}\limits_{k}\frac{{\tilde{g}}^{2k}{e}^{-ik2\pi {f}_{cav}t}}{k!}$$

21 $${\left\langle {X}^{{{{\dagger}}} }(0)X(t)\right\rangle }_{{\hat{H}}_{cav}}={e}^{-{\tilde{g}}^{2}}{e}^{{\tilde{g}}^{2}{e}^{i2\pi {f}_{cav}t}}={e}^{-{\tilde{g}}^{2}}\mathop{\sum}\limits_{k}\frac{{\tilde{g}}^{2k}{e}^{i2\pi {f}_{cav}t}}{k!}$$

By substituting Eqs. (18)–(21) into Eq. (11) we obtain the expression

22 $${I}_{C}=\frac{e}{\hslash }i{e}^{-{\tilde{g}}^{2}}\int\nolimits_{-\infty }^{+\infty }\frac{d\omega }{2\pi }\frac{{{{\Gamma }}}_{L}{{{\Gamma }}}_{R}}{{{{\Gamma }}}_{L}+{{{\Gamma }}}_{R}}\left(({f}_{L}(\omega )-{f}_{R}(\omega ))\mathop{\sum}\limits_{k,d}\frac{{\tilde{g}}^{2k}}{k!}\left({G}_{d}^{ \,{ > }\,}\right.(\omega -k{\omega }_{0})-{G}_{d}^{\,{ < }\,}(\omega +k{\omega }_{0})\right)$$

Above, the lesser and greater Green’s functions of the dot can be calculated by using the Keldysh description of quantum dot circuits as:

23 $${G}_{d}^{ \,{ > }\,}(\omega )=\frac{-i\left({{{\Gamma }}}_{L}(1-{f}_{L}(\omega ))+{{{\Gamma }}}_{R}(1-{f}_{R}(\omega ))\right)}{{(\omega -{\bar{\varepsilon }}_{d})}^{2}+{({{\Gamma }}/2)}^{2}}$$

and

24 $${G}_{d}^{\,{ < }\,}(\omega )={{{\Sigma }}}^{\gtrless }(\omega )\frac{i\left({{{\Gamma }}}_{L}{f}_{L}(\omega )+{{{\Gamma }}}_{R}{f}_{R}(\omega )\right)}{{(\omega -{\bar{\varepsilon }}_{d})}^{2}+{({{\Gamma }}/2)}^{2}}$$

with Γ = Γ_*L*_ + Γ_*R*_. We assume that the levels ${\bar{\varepsilon }}_{d}$ (or *ε*_*d*_) are separated by a level spacing Δ. We have used equations (22), (23) and (24) to plot Fig. 3d, e with Γ_*L*(*R*)_/*f*_*cav*_ = 0.1, $\tilde{g}=2$, *x* = 0.7, Δ/*f*_*cav*_ = 0.6 and *T*/*f*_*cav*_ = 0.05. For Fig. 3e we have used the same parameters and *ε*_*d*_/*f*_*cav*_ = 0.4. Note that for $\tilde{g}=0$, one recovers the standard expression of the current through an independent dot^34,35^, i.e.

25 $${I}_{C}=\frac{e}{\hslash }i\int\nolimits_{-\infty }^{+\infty }\frac{d\omega }{2\pi }\frac{{{{\Gamma }}}_{L}{{{\Gamma }}}_{R}}{{{{\Gamma }}}_{L}+{{{\Gamma }}}_{R}}\left(({f}_{L}(\omega )-{f}_{R}(\omega ))\mathop{\sum}\limits_{d}\left({G}_{d}^{ \,{ > }\,}\right.(\omega )-{G}_{d}^{\,{ < }\,}(\omega )\right)$$

Due to Eq. (22), for $\tilde{g}$ sufficiently large, the dot conductance shows steps which are due to photo-assisted tunneling for *eV* = ±*hf*_*cav*_, ±2*hf*_*cav*_, ±3*hf*_*cav*_,... and a current suppressed quadratically in ${(2\pi {g}_{l}/{f}_{cav})}^{2}$ for $e\left|V\right| < {f}_{cav}$ with small “subgap” resonances. When Δ ≫ *V*, *k*_*B*_*T*, Γ_*L*(*R*)_, and when a single dot level *d*_0_ is close to the reservoir Fermi energies for *V* = 0, the ratio between the subgap conductance *G*(*V* = 0) and the conductance *G*_1_ for *eV* = *hf*_*cav*_ + 0^+^ can be expressed in the limit Γ_*L*_ = Γ_*R*_ = Γ/2 as

26 $$\frac{G(V=0)}{{G}_{1}}\simeq {\tilde{g}}^{2}\frac{\left(1+\frac{{f}_{cav}^{2}}{{{{\Gamma }}}^{2}}\right)\left(1+2\frac{{f}_{cav}^{2}}{{{{\Gamma }}}^{2}}\right)}{1+4\frac{{f}_{cav}^{f}2}{{{{\Gamma }}}^{2}}}$$

with

27 $$G(V=0)\simeq \frac{2\exp (-{\tilde{g}}^{2}){{{\Gamma }}}_{L}{{{\Gamma }}}_{R}}{\pi \left({(\omega -{\bar{\varepsilon }}_{{d}_{0}})}^{2}+{({{\Gamma }}/2)}^{2}\right)}$$

From equation (22), for *T* = 0, *x* = 1/2 and Δ ≫ Γ, we can derive analytically the subgap conductance peak height to be $\approx \frac{{e}^{2}}{h}{e}^{-{\tilde{g}}^{2}}$ and a gap edge at *V* ≈ ± *f*_*cav*_ with a magnitude $\approx \frac{{e}^{2}}{h}{\tilde{g}}^{2}{e}^{-{\tilde{g}}^{2}}/2$. This allows us to get a ratio *R* between the latter and the former of $R\approx {\tilde{g}}^{2}/2$. From Fig. 3b, we estimate the experimental *R* to be 5 and therefore $\tilde{g}\approx 3\pm 0.5$. The error bars on $\tilde{g}$ correspond to the estimated systematic errors in determining the parameters of the quantum dot.

## Supplementary information

Supplementary Information

## Data Availability

The authors declare that the main data supporting the findings of this study are available within the article (main text, methods, and supplementary). Extra data are available from the corresponding author upon reasonable request.

## Code availability

The authors declare that the codes used for the work presented in the paper are available upon reasonable request.
