# Supplementary material for: Vacuum-field-induced THz transport gap in a carbon nanotube quantum dot
Source: Nat Commun. 2021 Sep 16;12:5490. doi: 10.1038/s41467-021-25733-x (PMC8446012; doi:10.1038/s41467-021-25733-x)
Supplement: Supplementary file 1 — Supplementary Information [file 41467_2021_25733_MOESM1_ESM.pdf]

Supplementary Information - Vacuum-field-induced THz transport gap in a carbon nanotube quantum dot

F. Valmorra[1], K. Yoshida[2], L.C. Contamin[1], S. Messelot[1], S. Massabeau[1], M.R. Delbecq[1], M.C. Dartiailh[1], M.M. Desjardins[1], T. Cubaynes[1], Z. Leghtas[1], K. Hirakawa[2], J. Tignon[1], S. Dhillon[1], S. Balibar[1], J. Mangeney[1], A. Cottet[1] and T. Kontos[1]

[1]*Laboratoire de Physique de l'Ecole normale supérieure, ENS, Université PSL, CNRS, Sorbonne Université, Université Paris-Diderot, Sorbonne Paris Cité, Paris, France and*

[2]*Institute for Nano Quantum Information Electronics, University of Tokyo, 4-6-1 Komaba, Meguro-ku, Tokyo 153-8505, Japan*

(Dated: August 27, 2021)

Abstract

We show in the supplementary information part some back-up material to further substantiate the main results presented in the main text. This includes additional data for devices A, B and C, Coulomb diamonds for a device without THz resonator and a discussion of the coupling mechanism and coupling strength.

I. SUPPLEMENTARY NOTE 1: MAPS FOR DEVICES A,B AND C

In this section, we present $V_{sd} - V_g$ maps of the conductance of samples A, B and C. It also shows one extra $G(V_{sd})$ curve for sample B. Supplementary Fig. 1 shows the $V_{sd} - V_g$ conductance map of Sample A already shown in Figure 3a, but on a larger scale. Supplementary Fig. 2 and 3 show the $V_{sd} - V_g$ conductance maps of Samples B and C whose $G(V_{sd})$ cuts are shown in panels 3b and 3c respectively. The arrows on the top of these three figures indicate the position of the $G(V_{sd})$ cuts shown in Figure 3b and 3c of the main text and Supplementary Fig. 4. The dashed horizontal lines indicate the position of the conductance steps/resonances expected at $eV_{sd} = -2hf_{cav}$, $eV_{sd} = -hf_{cav}$, $eV_{sd} = hf_{cav}$, and $eV_{sd} = 2hf_{cav}$. The line $eV_{sd} = 0$ is also shown in Supplementary Fig. 3. For Sample A (Supplementary Fig. 1), we have shifted vertically these lines at $V_g \simeq 200$ mV to take into account an offset in V_{sd}.

For sample A, a gap delimited by $eV_{sd} = \pm hf_{cav}$ is clearly visible in the conductance along the whole gate voltage range of Supplementary Fig. 1, as well as a conductance variation for at $eV_{sd} = \pm 2hf_{cav}$. For Sample B, a gap delimited by $eV_{sd} = \pm hf_{cav}$ is visible only in the range 20 mV $\lesssim V_g \lesssim$ 20 mV. This gap is less visible for the other gate voltage ranges. One can also guess the presence of slight conductance steps at $eV_{sd} = \pm 2hf_{cav}$ in some areas of the the figures. The variations in the visibility of these features can be attributed to the fact that when the dot gate voltage is varied, the spatial profile of the electronic Green's function changes, and consequently, the amplitude of the electron/photon coupling changes notably. Note that for sample B, negative differential resistance is indicated in green. Such a feature can already happen in the absence of electron/photon coupling and is often due to electronic interaction effects, but its occurrence may also be influenced/modified by the presence of the light/matter interaction. In sample C, the low voltage conductance gap is clearly delimited by a step at $eV_{sd} = hf_{cav}$, but the step at $eV_{sd} = -hf_{cav}$ is missing and seems to be replaced by a smooth limit at $eV_{sd} = 0$. In fact, a Kondo conductance ridge at $eV_{sd} = 0$ is even visible for 50 mV $\lesssim V_g \lesssim$ 65 mV. Therefore one can say that there is only a half gap in the data at 0 mV $\lesssim V_{sd} \lesssim hf_{cav}/e$, in agreement with the data shown in Fig. 3c of the main text.

Supplementary Fig. 4 shows one extra $G(V_{sd})$ curve for sample B, for $V_g = 13.8$ mV. An area with negative differential resistance ($G < 0$) is visible in this curve.

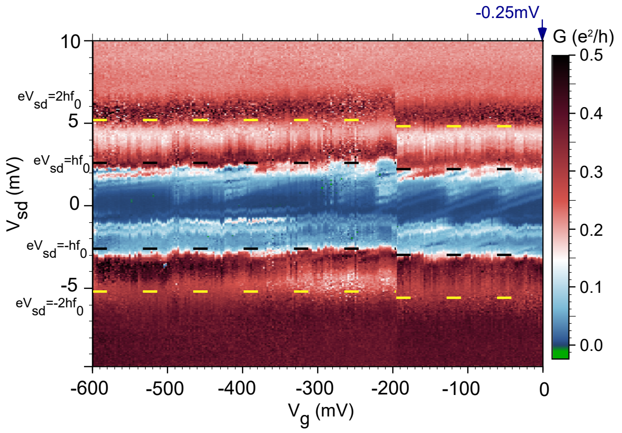

Supplementary Figure 1. Large conductance map for sample A Conductance colorscale plot of sample A shown on a wider gate scale than in the main text.

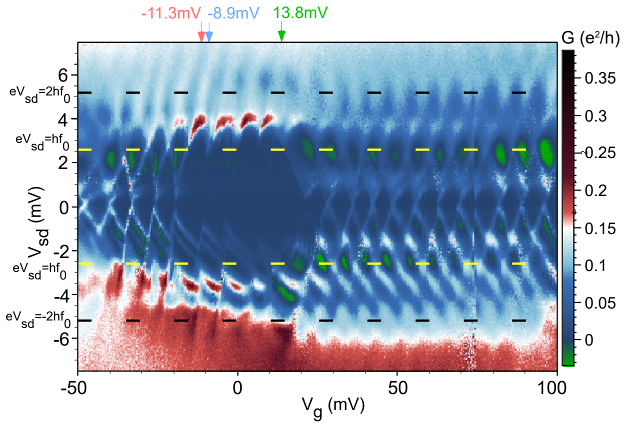

Supplementary Figure 2. Conductance map for sample B Conductance colorscale plot of sample B showing the regions in gate voltage where the gap is prominent.

II. SUPPLEMENTARY NOTE 2: DEVICE WITHOUT THZ CAVITY

We show in this section the conventional spectrum of a carbon nanotube quantum dot with similar dimensions as the ones studied in the present work. This nanotube is coupled weakly to a GHz resonator so we do not expect any feature occurring close to $\pm 2.6meV$. This is indeed the case as shown in Supplementary Fig. 5. We observe conventional Coulomb diamonds which close at zero bias and no horizontal stripe of conductance suppression is observed anywhere in the spectrum of the device (we show only a small part of the Coulomb blockade pattern for the sake of clarity).

The Coulomb blockade pattern observed for the control device is different than the other

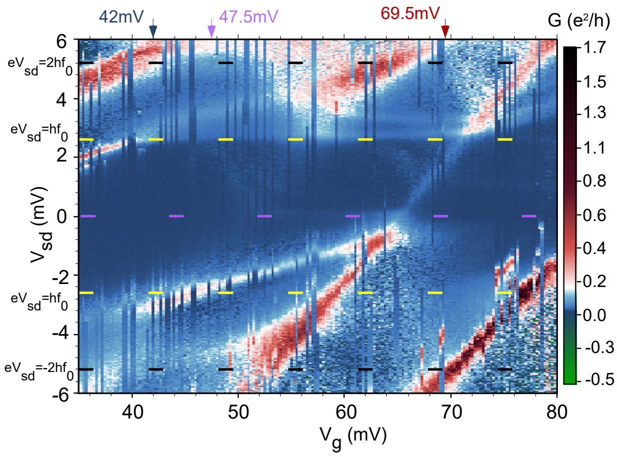

Supplementary Figure 3. Conductance map for sample C Conductance colorscale plot of sample C showing the regions in gate voltage where the gap is prominent.

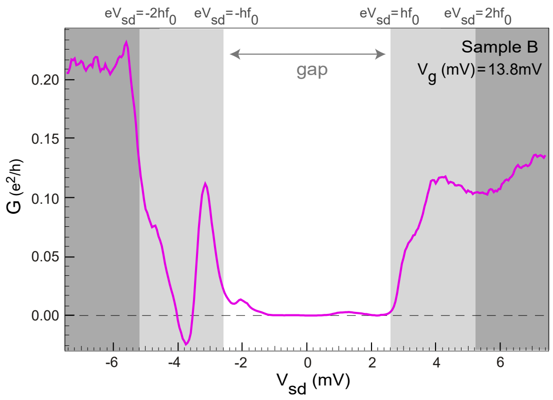

Supplementary Figure 4. Cut of conductance map for sample B Cut of the conductance map for sample B at $V_g = 13.8mV$. Negative differential conductance is observed in this example, but the gap is also present in this case.

Coulomb blockade patterns for devices A, B and C. As expected for many quantum dot platforms, there is an inherent variability in the nanotube quantum dots which arises from weak disorder. This leads to changes in the low energy spectrum of the nanotube quantum dot. This explains why the Coulomb blockade diamonds look very different in devices A, B and C. Interestingly, in all these 3 quantum dot circuits, we can identify the energy of the LC-mode in the spectrum at 2.6 meV despite the variability of the rest of the spectrum. This is one of the strongest arguments in favour of the deep strong coupling scenario in our devices.

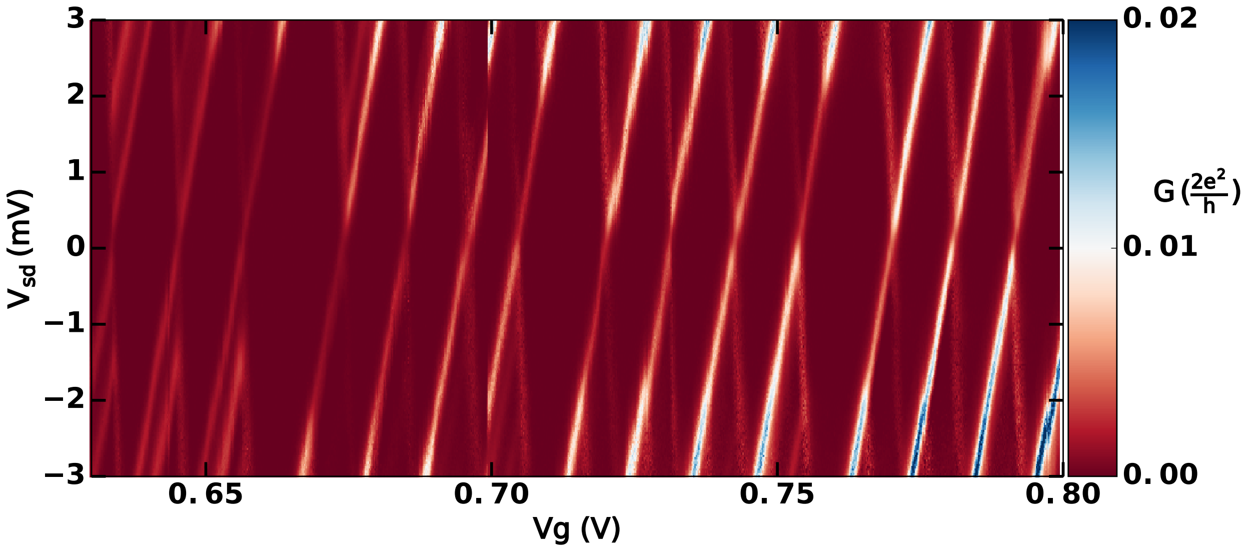

Supplementary Figure 5. Conductance map for a carbon nanotube single quantum dot with no THz cavity. There is no indication of any THz gap in this device.

III. SUPPLEMENTARY NOTE 3: DISCUSSION ON THE COUPLING STRENGTH

The usual definition of the coupling strength in cavity quantum electrodynamics is linked to the zero point fluctuations of the electric field $\mathcal{E}_{zpf}$(here we focus on the electric coupling as stated in the main text) which reads[1]:

$$\mathcal{E}_{zpf} = \sqrt{\frac{hf_{cav}}{\varepsilon_0 V_{cav}}} \tag{1}$$

where V_{cav} is the mode volume and ε_0 is the vacuum permittivity. This is a simplified expression which holds for atoms in vacuum and this is why we used the COMSOL and HFSS softwares to estimate $\mathcal{E}_{zpf}$ and the corresponding V_{zpf} in our case. The result of the HFSS simulation is shown in Supplementary Fig. 6. However, we can get interesting insights about the basic ingredient which help to boost the vacuum field fluctuations by inspecting the above formula. As shown in the main text, one can bridge between circuit QED to cavity QED and a reliable estimate of the coupling strength in our case is $g_l \approx e\mathcal{E}_{zpf}d$, where d is the typical size of the dipole which spans from below the THz gate to the source electrode, and e is the elementary charge. The quantity $C_{cav-matter} = \varepsilon_0 V_{cav}/d^2$ has the dimension of the a capacitance which is characteristic of how the dipole fits in the mode volume once

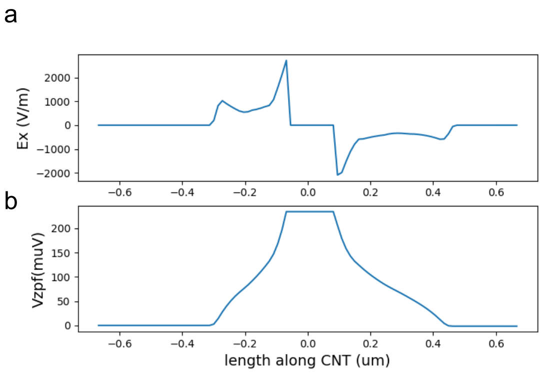

Supplementary Figure 6. Microwave simulation of the zero point fluctuation profile using HFSS. a. The electric field profile as a function of distance. **b.** The voltage profile as a function of distance.

both system are assembled. Defining the joint charging energy $E_{cav-matter} = e^2/C_{cav-matter}$, we arrive at an insightful expression :

$$g_l \approx V_{zpf} \approx \sqrt{hf_{cav}E_{cav-matter}} \tag{2}$$

This expression should of course take into account the electron-electron interactions which are expected to modify both the mode volume and the size of the dipole due to their role in the screening of the electric field. For typical sizes of dipoles and cavity modes, this charging energy can be very large, as large as the one measured for the nanotube i.e. in the several meV range. This suggests that the deep strong coupling limit may be reached for nanoscale conductors with large interaction effects. In our case, both COMSOL and HFSS show that already without the nanotube, V_{zpf} can be in the $200 - 400\mu V$ range. Electron-electron interactions play a crucial role in screening processes. Our findings suggest that they could boost $\tilde{g}$ further into the deep strong coupling regime.

[1] C. Cohen-Tannoudji, J. Dupont-Roc and G. Grynberg, *Photons and Atoms*, Wiley (1997).
